# Internalization of Pegylated Er:Y_2_O_3_ Nanoparticles inside HCT-116 Cancer Cells: Implications for Imaging and Drug Delivery

**DOI:** 10.1021/acsanm.3c03609

**Published:** 2023-10-05

**Authors:** Regina Maria Chiechio, Angela Caponnetto, Rosalia Battaglia, Carmen Ferrara, Ester Butera, Paolo Musumeci, Riccardo Reitano, Francesco Ruffino, Giuseppe Maccarrone, Cinzia Di Pietro, Valérie Marchi, Luca Lanzanò, Giovanni Arena, Alfina Grasso, Chiara Copat, Margherita Ferrante, Annalinda Contino

**Affiliations:** † Dipartimento di Fisica e Astronomia “Ettore Majorana”, Università di Catania, Via Santa Sofia 64, 95123 Catania, Italy; ‡ Consiglio Nazionale delle Ricerche, Istituto per la Microelettronica e i Microsistemi (CNR-IMM), Via S. Sofia 64, 95123 Catania, Italy; § Dipartimento di Scienze Biomediche e Biotecnologiche, Sezione di Biologia e Genetica “G. Sichel”, Università di Catania, Via S. Sofia 89, 95123 Catania, Italy; ∥ Dipartimento di Scienze Chimiche, Università di Catania Viale Andrea Doria 6, 95125 Catania, Italy; ⊥ Institut des Sciences Chimiques de Rennes, CNRS UMR 6226, Université Rennes 1, Avenue du général Leclerc, 35042 Rennes, France; # Environmental and Food Hygiene Laboratories (LIAA) of Department of Medical, Surgical Sciences and Advanced Technologies “G.F. Ingrassia”, University of Catania, 95124 Catania, Italy

**Keywords:** Nanoparticles, luminescence, lanthanides, pegylation, cancer cells, internalization

## Abstract

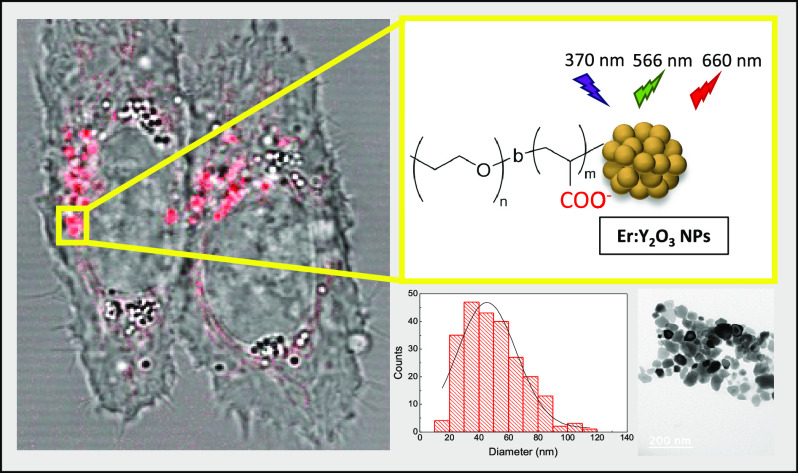

Lanthanide-doped nanoparticles, featuring sharp emission peaks with narrow bandwidth, exhibit high downconversion luminescence intensity, making them highly valuable in the fields of bioimaging and drug delivery. High-crystallinity Y_2_O_3_ nanoparticles (NPs) doped with Er^3+^ ions were functionalized by using a pegylation procedure to confer water solubility and biocompatibility. The NPs were thoroughly characterized using transmission electron microscopy (TEM), inductively coupled plasma mass spectrometry (ICP-MS), and photoluminescence measurements. The pegylated nanoparticles were studied both from a toxicological perspective and to demonstrate their internalization within HCT-116 cancer cells. Cell viability tests allowed for the identification of the “optimal” concentration, which yields a detectable fluorescence signal without being toxic to the cells. The internalization process was investigated using a combined approach involving confocal microscopy and ICP-MS. The obtained data clearly indicate the efficient internalization of NPs into the cells with emission intensity showing a strong correlation with the concentrations of nanoparticles delivered to the cells. Overall, this research contributes significantly to the fields of nanotechnology and biomedical research, with noteworthy implications for imaging and drug delivery applications.

## 1 Introduction

Nanomaterials have recently become one of the most interesting research areas in chemistry, biotechnology, and biomedicine. In fact, it has been demonstrated that inorganic nanomaterials, thanks to their significant properties such as biocompatibility, ease of synthesis, and ease of surface functionalization, show great potential in bioimaging, targeted drug delivery, and cancer therapies.^1−3^

Bioimaging is a noninvasive technique for visualizing biological processes, allowing observation of subcellular structures, cells, tissues, and complete multicellular creatures.^4^ In particular, fluorescence bioimaging makes it possible to study many cellular phenomena in situ, accurately monitoring the state of cells in vivo and managing to highlight the presence of biomarkers and biochemical indicators of specific processes.^5^ In addition, tumor bioimaging represents a noninvasive, rapid, and very sensitive method for the early diagnosis of tumors, as it allows the visualization and localization of cancerous and precancerous tissues.^6,7^ Following the high interest in the dynamics of intracellular components in living cells, the imaging of regions inside the cell using electron microscopy^8^ and confocal microscopy^8,9^ has become of extreme importance to the scientific community;^10^ this need has led to the development of new probes, more stable and performing. In this context, fluorescent nanoparticles (NPs) have been widely used in the past decade for fluorescence imaging. Their use for imaging and drug delivery is one of the most interesting and important areas in biomedicine. They can be engineered to acquire peculiar and unique properties, that can provide novel tools and techniques in biomedical research. A deeper investigation on interactions between NPs and biological systems is necessary in developing effective NPs as sensing, imaging and drug delivery agents.^11^

While organic dyes often show poor photostability, fluorescent nanomaterials offer the advantages of a high quantum yield, a large Stokes shift, but above all high photostability and chemical stability.^12−14^ For this reason, different types of fluorescent nanomaterials, including semiconductor quantum dots,^15^ noble metal nanoparticles,^16,17^ and carbon nanomaterials,^18^ have been explored. In recent decades, research has begun to focus on the use of rare earth nanoparticles, whose interesting optical properties, such as narrow emission bandwidths and high resistance to photobleaching,^19^ high chemical stability, and low cytotoxicity,^20^ make them excellent candidates for biological applications. Usually, lanthanide ions such as Er^3+^ with high and very narrow emissions are used as dopant for Y_2_O_3_, a very good matrix that, owing to its thermal stability, can be treated at very high temperature allowing the obtainment of compounds with a high degree of crystallinity and thus better photoluminescence properties.^21^ Above all, rare earth ions have upconversion luminescence properties, whereby if irradiated in the far NIR they can emit in the near NIR or in the visible. Since NIR light has lower absorption and scattering in biological tissues, thus achieving high penetration efficiency, fluorescence imaging technology mainly makes use of the NIR window.^22−26^ Thus, lanthanide-doped yttrium oxide nanoparticles, exhibiting upconversion properties, are prime candidates for in vivo bioimaging.^13,19,27^ Y_2_O_3_ nanoparticles, due to the chemical inertness of this material, are also characterized by a not high toxicity which makes them widely used in the biological field.^28−31^ These systems, however, being “ceramic” are insoluble in an aqueous medium, and it is unlikely that they could be able to penetrate into the cells, as they are. It is therefore necessary to suitably functionalize them in order to reduce their toxicity and to use them for biological applications. Coating the surface of nanoparticles with polyethylene glycol, known as “PEGylation”, is a common approach to make systems more soluble and to reduce capture by the reticuloendothelial system.^32,33^ This process definitely improves the circulation in bloodstream, the internalization,^34^ and the accumulation at tumor sites.^35^ It is also extremely important to use nanoparticles of the appropriate size to facilitate internalization inside cells. Indeed, particles that are too large may not be able to overcome the lipid bilayer and enter the cytoplasm. In the particular case of rare earths-doped Y_2_O_3_ nanoparticles, however, it was noted that too small particles give lower luminescence signals.^36,37^ Recently, some of us have developed a synthetic procedure^38^ by which it is possible to obtain NPs with a fairly high yield and suitable dimensions to simultaneously optimize the fluorescence intensity and the ability to penetrate inside the cells.

A careful evaluation of effects such as toxicity induced by different types of NP formulations in biological systems is important in employing NPs for biological applications. One of the central features in nanomedicine is the controlled interaction of NPs with target cells, in order to overcome physical and chemical limits and avoid undesired toxicity in the long term.^39^ Novel technologies for in vitro testing and innovative computational methods are sought, enabling the prediction of NPs’ interactions with living organisms, commencing from cellular models.

This work investigates the internalization of pegylated erbium-doped Y_2_O_3_ nanoparticles (Er:Y_2_O_3_) in human colorectal cancer cells (HCT-116) assessing the nontoxic concentration for cell viability which gives rise to a detectable fluorescence signal and confirming their presence inside cells by confocal microscopy and ICP-MS analyses. In this respect, ICP-MS provided a very accurate and selective quantification of the internalized nanoparticles, allowing us to single out the contribution due solely to the nanoparticles. Thus, this study allowed the obtainment of a nanomaterial with important implications for imaging and drug delivery.

## 2 Materials and Methods

### 2.1 Materials

Y(NO_3_)_3_·6H_2_O, Er(NO_3_)_3_·5H_2_O, and urea were obtained as commercial reagents by Alfa Aesar (USA). Urease and polyacrilic acid (PAAc) were purchased from Sigma-Aldrich (USA), whereas PAAc-*b*-PEG (*M*_n_ = 5000/3200) was purchased from Polymer Source, Inc. RPMI 1640 medium, fetal bovine serum (FBS), and streptomycin/penicilin (10,000 U/mL) were obtained by Gibco, Thermo Fisher Scientific, Waltham, MA., l-glutamine from Lonza (Basel, Switzerland), and dimethyl sulfoxide (DMSO) from PanReac AppliChem. Phosphate buffered saline (PBS) was purchased from Merck (Milan, Italy) and 0.05% trypsin and 0.53 mM EDTA (1X) from Corning, Mediatech, Inc., Manassas (USA). For ICP measurements, DigiPREP SCP SCIENCE was obtained from Clark Graham Baie D’Urfé, Quebec (Canada). The nitric acid for trace analysis was obtained from HNO3-Carlo Erba (Italy), and the Rh stock solution was obtained from CPAChem (Bulgary). All solutions were prepared using Milli Q water. HCT-116 cell lines, derived from primary colon tumors, were obtained from the Interlab Cell Line Collection (ICLC), an “International Repository Authority” within the IRCCS Azienda Ospedaliera Universitaria San Martino-IST Istituto Nazionale per la Ricerca sul Cancro (Genova, Italia).

### 2.2 Synthesis of Er:Y_2_O_3_ Nanoparticles

The nanoparticles were synthesized by slightly modifying the procedure previously reported by Venkatachalam et al.^40^ by using nitrates instead of carbonates.^38^ In this case, in the starting mixture (400 mL) composed of urea (0.4 mol), nitrates of yttrium (4 mM) and erbium (0.3 mM), and PAAc (0.1 mM) was kept at 90 °C for 1 h. Then, the precipitate precursors were separated by centrifugation and washed three times (5000 rpm for 15 min each time) with Milli-Q water and dried at 100 °C for 12 h. The dried precursor materials were calcined in an air atmosphere at 1100 °C for 60 min to improve the crystallinity of the final product and finally crushed in an Agate mortar to make them homogeneous.

### 2.3 Preparation of PEGylated Er:Y_2_O_3_ Nanoparticles

The pegylation of Er:Y_2_O_3_ nanoparticles was carried out by slightly modifying the procedure reported by Kamimura et al.^41^ Briefly, to 20 mL of a buffer solution TRIS/HCl (pH 7.00), containing 0.5 g/L of PEG-*b*-PAAC, 2 mg of nanoparticles were added, and the resulting mixture was kept at 4 °C for 24 h under magnetic stirring. The obtained solution was purified by ultracentrifugation (9.0 × 10^4^ g, 15 min, 3 times), and the solvent was changed to Milli-Q water.^38^

### 2.4 TEM Analyses

Transmission electron microscopy analysis was carried out with a JEOL 1400 transmission electron microscope (Japan). For the sample preparation, 300 mesh carbon coated nickel grids were placed for 1 min on top of a 40 μL sample droplet (colloidal dispersion of pegylated Er:Y_2_O_3_ NPs) and dried with paper. A 200 kV acceleration voltage was used. Particles distributions were determined from TEM micrographs using ImageJ software.

### 2.5 Photoluminescence

Photoluminescence measurements were performed on a Horiba Nanolog spectrofluorometer (France). The measurements were performed at room temperature on samples drop-cast on silicon (three drops). The wavelength resolution of both the excitation and the emission slits was set to 5 nm for measurements in the visible range and 14 nm for measurements in the IR range. The intrinsic fluorescence of pegylated Er:Y_2_O_3_ nanoparticles was excited at 378 nm, and the corresponding emission spectra were acquired both in the visible and IR region, by using a photomultiplier Hamamatsu R928 in the UV–vis-NIR and a Horiba Symphony II InGaAs array in the IR detector, respectively. Two different long-pass filters (400 nm for the visible range and 840 nm for the IR) were used in order to block the lamp excitation wavelength. The acquisition times were 1 s for measurements in the visible range and 60 s for measurements in the IR range.

### 2.6 Cell culture

Human colorectal cancer cells (HCT-116) were cultured in RPMI 1640 medium supplemented with 10% FBS, 1% streptomycin/penicilin (10,000 U/mL), and 2 mM l-glutamine. Cells were cultivated at 37 °C and 5% CO_2_.

### 2.7 Cell Viability Assay

HCT-116 cells were seeded at 37 °C and 5% CO_2_ in a 96-well plate at a density of 1.5 × 10^4^ cells per well and starved after 24 h of seeding. Then, cells were exposed to different concentrations of Er:Y_2_O_3_ NPs (0.1, 0.25, 0.5, and 1 μg/mL) at different time points (24–48 h) at 37 °C with 5% CO_2_. Cell survival rate was assayed by the 3-(4,5-dimethyl-2-thiazolul)-2,5-diphenyl-2H-tetrazolium bromide assay (MTT) (Millipore). Briefly, 10 μL of MTT plus 90 μL of RPMI 1640 were added to each well except for the cell-free blank wells. Cells were incubated for 4 h at 37 °C with 5% CO_2_. After 4 h of incubation, the MTT solution was removed and replaced with 100 μL of DMSO, and the plates were further incubated for 20 min at room temperature. The optical density of the wells was determined using a VariosKan (Thermo Fisher Scientific, Waltham, MA) plate reader at a test wavelength of 595 nm and a reference wavelength of 655 nm. All experiments were performed in four biological replicates. Statistical analysis was performed applying an unpaired *t* test, and statistical significance was assessed by setting the p-value cutoff ≤ 0.05.

### 2.8 ICP Analyses

HCT-116 cells were seeded at 37 °C and 5% CO_2_ in a 96-well plate at a density of 1.5 × 10^4^ cells per well in four replicates and starved after 24 h of seeding. Then, cells were exposed to different concentrations (0.1, 0.25, 0.5, and 1 μg/mL) of Er:Y_2_O_3_ nanoparticles (NP). Yttrium (Y) and erbium (Er) were quantified both in the NPs alone and with the cells to verify the internalization process. More in detail, after 24 h of treatment, cells exposed to Er:Y_2_O_3_ NPs and not exposed (used as negative control) were washed three times with 100 μL of water in order to remove the not internalized NPs, detached from the plate with 50 μL of 0.05% trypsin, 0.53 mM EDTA (1X), and 100 μL of complete medium. The four replicates were pulled together and centrifuged (Beckman centrifuge, J-6M/E, JS 5.2 rotor). The supernatant was removed, and the cellular pellets were washed with 100 μL of water by centrifugation. All the centrifugations were performed at 1100 rpm for 15 min at 20 °C.

Samples were digested using a DigiPREP SCP SCIENCE by adding to 1 mL of suspended NPs or 0.020 g of cell pellet 2 mL of 65% nitric acid for trace analysis and 1 mL of ultrapure water in HDPE vessels over an operating cycle of 1 h at 120 °C. Analytical blanks were analyzed in the same way as the samples. The vessels were allowed to cool, and then, the sample volume was made up to 10 mL with ultrapure water. An ICP-MS Elan-DRC-e (PerkinElmer, USA) was used for quantification of total Y and total Er. Sample concentrations were determined using a linear through zero interpolating function with a calibration blank and six standard solutions (ranging from 1 to 100 μg/l) prepared in the same sample acid matrix. Both calibration standards and samples were spiked to a final concentration of 25 μg/L using an Rh stock solution (10 mg/L Rh in 2% nitric acid) as an internal standard. To monitor the matrix effect and significant changes in instrumental sensitivity, the intensity of the internal Rh standard was monitored in each analysis, using as a criterion an Rh intensity of 70%–130% in the sample analysis versus the Rh intensity in the calibration blank. At the end of calibration, every 10 samples, and at the end of the analytical series, a control standard were determined. Each test standard agreed with ±10% of the nominal concentration of Y and Er of 10 μg/L. Results were expressed in μg/g units for cell pellet samples and in mg/L for Er:Y_2_O_3_ NP suspensions.

### 2.9 Confocal Microscopy

HCT-116 cells were seeded at 37 °C and 5% CO_2_ on chambered coverslips (μ-slide 8 well glass bottom, Ibidi, Germany) at a density of 4.5 × 10^4^ cells per well following the same culture and NPs exposition conditions of viability assay, with only few differences. In particular, after both 24 and 48 h of NPs exposition, cells were washed with PBS solution, and new fresh culture medium was added for microscopy measures. Confocal fluorescence images were acquired on a Leica TCS SP8 confocal microscope using an HCX PL APO CS2 63X 1.40 NA oil immersion objective lens (Leica Microsystems, Mannheim, Germany). Excitation was provided by a laser source at 488 nm, and the emission was detected in the band 545–580 nm using a hybrid photodetector. The pinhole size was set to 1 Airy Unit. Transmitted light images were acquired simultaneously using the transmitted light detector (TLD) to visualize the morphology of the cells. During imaging, cells were kept at 37 °C and 5% CO_2_. Confocal spectral images were acquired by using the λ-scan mode of the Leica TCS SP8 confocal microscope. To acquire these images, the detection band was set to 10 nm and scanned from 500 to 788 nm with a step of 6 nm. These images were used to measure the emission spectra in different areas of the sample. The confocal fluorescence images were analyzed by choosing eight tiled square regions of the same dimensions in the zones of greatest intensity for each concentration. The intensity of the control was subtracted from the average luminescence intensity of the eight regions.

## 3 Results and Discussion

### 3.1 Synthesis and Characterization of Pegylated Nanoparticles

**Scheme 1:**
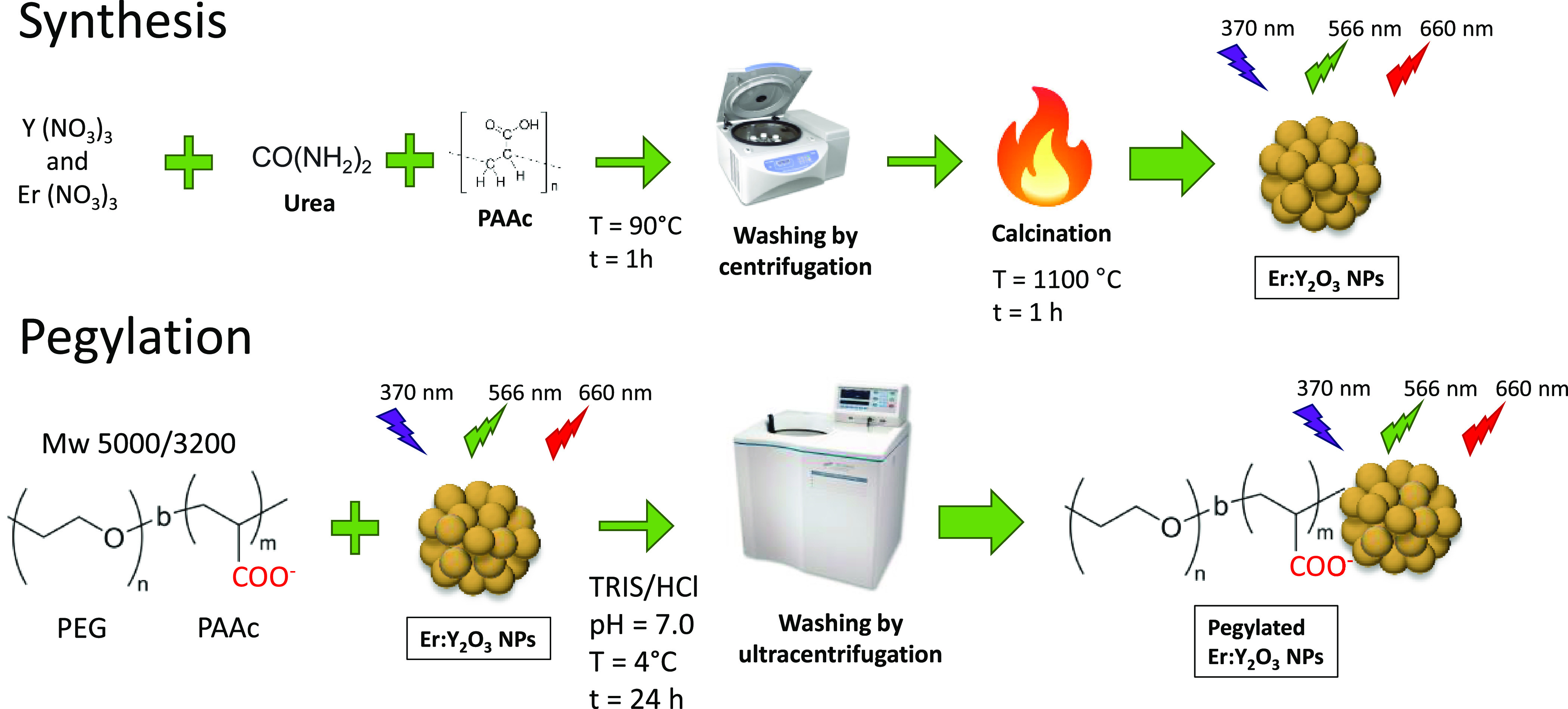
Schematic Description of Synthesis and Pegylation Processes

The Er-doped Y_2_O_3_ nanoparticles were synthesized as previously reported,^38,40^ by precipitating the erbium and yttrium hydroxides in a basic environment and using PAAc as template in order to obtain particles with the dimensions as smallest as possible. The obtained precipitate consisting of hydroxides was calcinated at high temperatures either to remove the organic residues and water or to increase the crystallinity of the nanoparticles (NPs) (Scheme 1). Maintaining a high rate of crystallinity is crucial for this type of NPs, since the Er present inside emits by stark effect only if it is located within a well-ordered crystal lattice.^42−45^

However, since the Y_2_O_3_ particles are insoluble in water, it was necessary to proceed with a pegylation process in order to reduce aggregation and increase the affinity with water. This process is also essential for biological applications, since PEG prevents interactions with other elements present in the blood flow which, through the “protein corona effect”, would increase the size of the NPs making them more difficult to eliminate from the body as well as more visible to the immune system, decreasing circulation time. For pegylation, a block copolymer called PEG-*b*-PAAc was used.^41^ This polymer is able to electrostatically interact with the NPs surface through the carboxylate groups of the PAAc block, whereas exposing the neutral part of the PEG toward the outside (Scheme 1).

**Figure 1:**
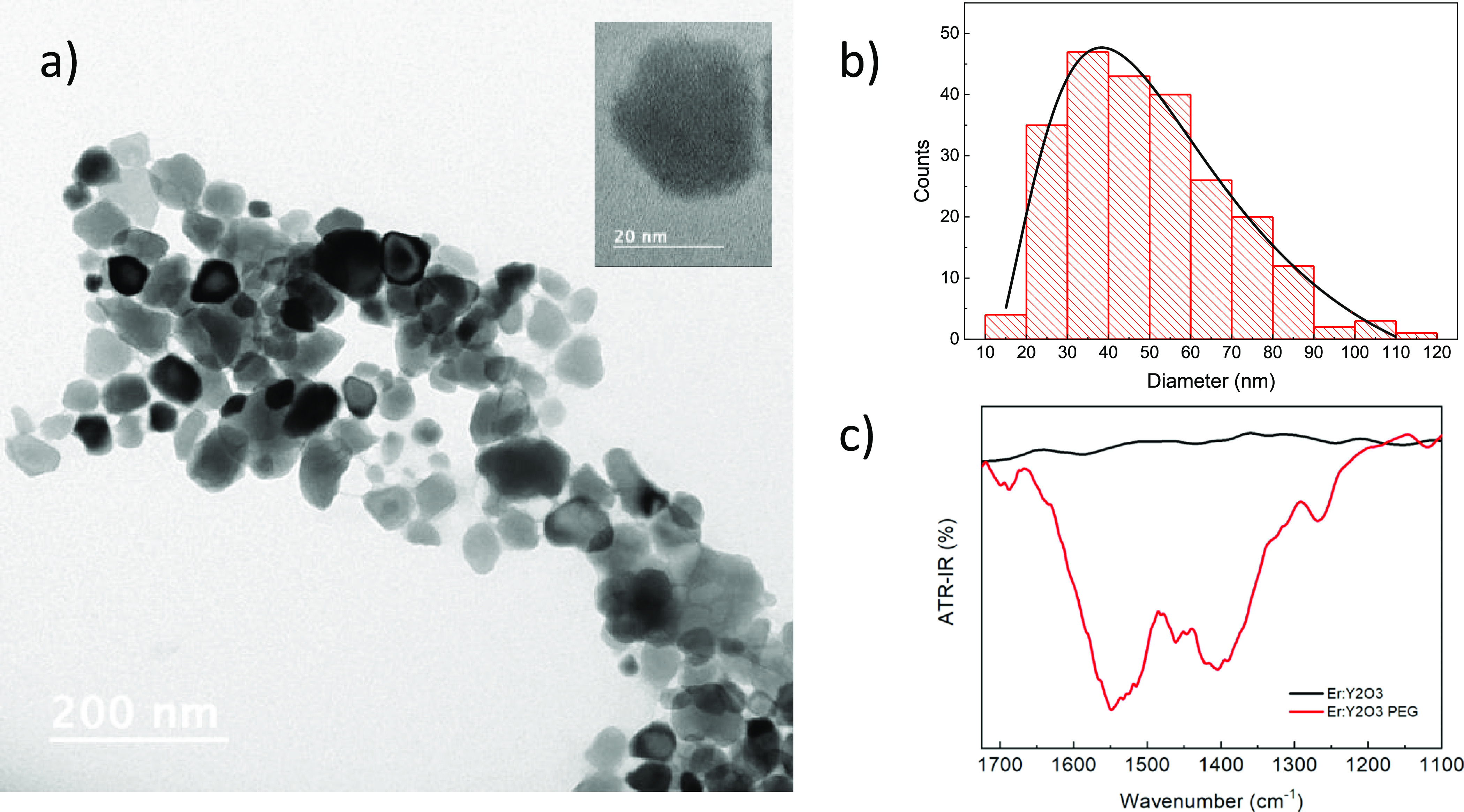
(a) TEM microphotograph, (b) related particle size distribution for pegylated Er:Y_2_O_3_ nanoparticles, and (c) FT-IR spectra of bare (black line) and pegylated (red line) Er:Y_2_O_3_ nanoparticles.

The HR-TEM microscopies of the pegylated Er:Y_2_O_3_ nanoparticles are reported in Figure 1a. The particles are well separated from each other because of the presence of the polymer and show a faceted spheroidal shape. The data were fitted using the log-normal distribution:
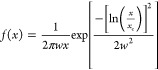
with *w* = width of the distribution, and *x*_c_ = median particle diameter. The fit (black line) in Figure 1b yields *w* = 0.58 ± 0.07, and *x*_c_ = 53.6 ± 4.1 nm. The most representative particle size corresponding to the fit maximum value is 37.9 nm.^46^ The apparent aggregation phenomenon is due to the drying process on the grid; however, the nanoparticles are not actually in contact with each other thanks to the presence of PEG. In the inset, it is also possible to notice the presence of crystalline planes showing a high crystallinity of the NPs.

The FT-IR spectra of bare and pegylated Er:Y_2_O_3_ nanoparticles are reported in Figure 1c. The pegylated NPs spectrum shows two characteristic absorption bands at 1550 and 1400 cm^–1^, typical of −COO^–^ stretches that are not present in the bare nanoparticles spectrum, confirming the surface modification.^47^

To evaluate the percentage of Er present in the NPs, ICP-MS measurements were carried out. The Er/Er + Y value found (0.09) was slightly higher than the expected one (Er/Er + Y = 0.07) and essentially the same to that previously obtained,^38^ that is the best to obtain a high fluorescence signal.^48,49^

Before being used for internalization studies with cells, the optical properties of the particles were probed in order to verify that the luminescence characteristics were maintained even after the pegylation process. To perform these measurements, the NPs solution was deposited and dried on a silicon support to increase the concentration and have a stronger signal. The NPs were excited using a wavelength (378 nm) corresponding to the maximum of the photoexcitation curve,^38^ in order to have a maximum emission signal.

**Figure 2:**
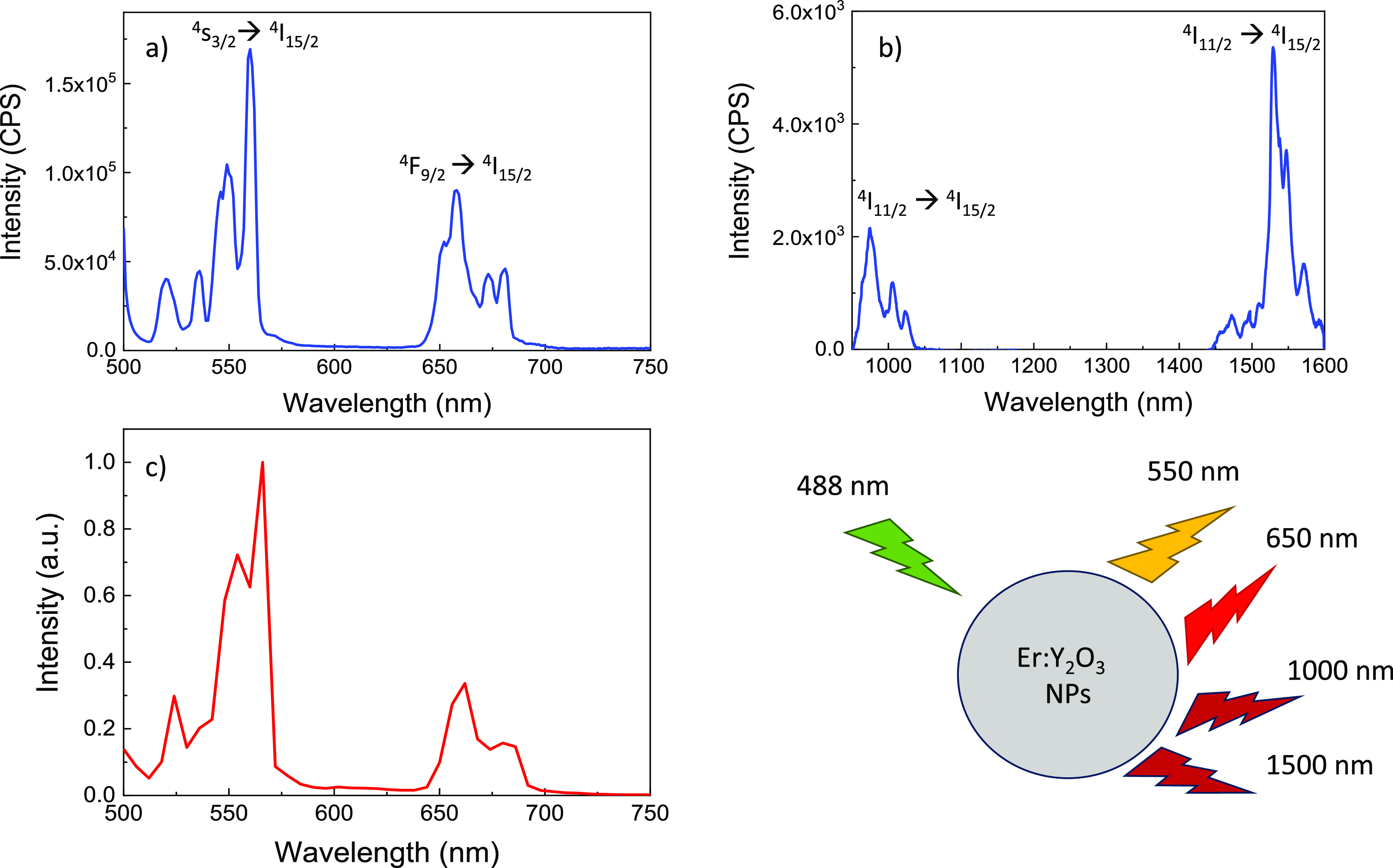
PL spectra of bare Er:Y_2_O_3_ NPs in the visible (a) and IR (b) range. (c) Confocal microscopy luminescence of pegylated Er:Y_2_O_3_ NPs (λ_exc_ = 488 nm).

The PL spectra for the pegylated nanoparticles are reported in the visible and IR ranges in Figure 2a and b, respectively. The typical peaks of erbium are present in the visible region at approximately 550 and 660 nm, and in the infrared region at 980 and 1522 nm, clearly indicating that, after the PEGylation, the nanoparticles core still maintained itsluminescence properties with satisfactory intensities; in particular, the same transitions of the naked nanoparticles were observed.^38^ This behavior is due to the fact that the crystalline characteristics of the material are not significantly altered.^50,51^

The PL spectrum of the nanoparticles in solution obtained by a confocal microscope is reported in Figure 2c. The spectrum shows the same peaks as those observed in Figure 2a.

### 3.2 Pegylated Nanoparticles Toxicity and Internalization inside HCT-116 Cancer Cells

**Figure 3:**
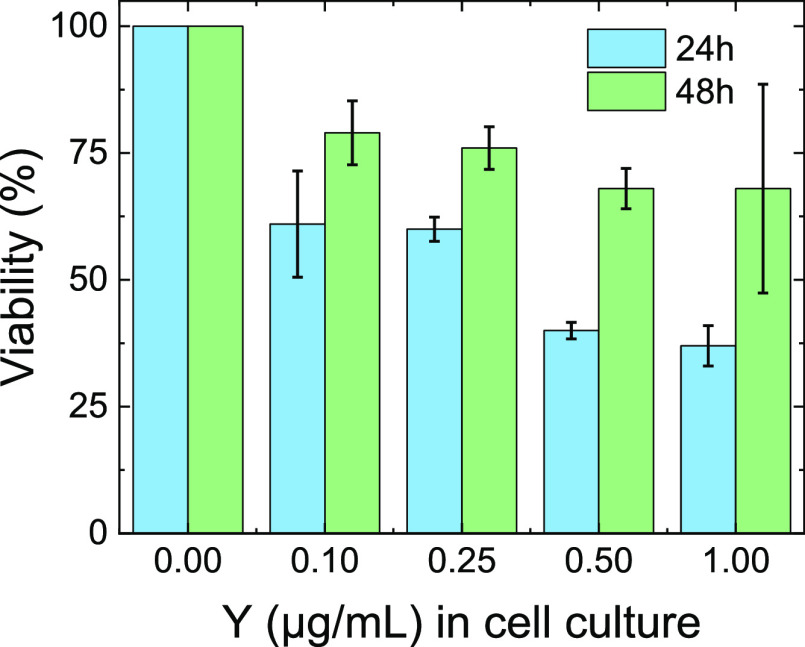
Viability percentage (%) of HCT-116 incubated with pegylated Er:Y_2_O_3_ nanoparticles at different concentrations: blue bars at 24 h and green bars at 48 h. Only the majority component (yttrium) was used to define the concentration of nanoparticles in the solutions administered to the cells. Values are reported as mean ± SD; *n* = 4. All data showed a p-value *<* 0.05 vs control.

An MTT assay was carried out after incubation of HCT-116 cells with Er:Y_2_O_3_ NPs at different concentrations (0.1, 0.25, 0.5, and 1 μg/mL) and at two incubation times (24 and 48 h). The results, reported in Figure 3, show that Er:Y_2_O_3_ NPs significantly influenced cell viability in a dose-dependent manner; 60% of viability was obtained at NP concentrations of 0.1 and 0.25 μg/mL at 24 h, and almost 80% of viability at 48 h at the same concentrations (Figure 3). The data indicate that, at 48 h, the vitality of the cells is partially restored. Even if the 1 μg/mL concentration is a toxic one, it was possible to use a lower concentration (0.25 μg/mL) that is less toxic, but which gives rise to a still detectable signal. Probably, the pegylation allowed us to obtain a slightly better internalization process, and thus, if one side led to a better intake, the other side gave rise to a major toxicity.

**Figure 4:**
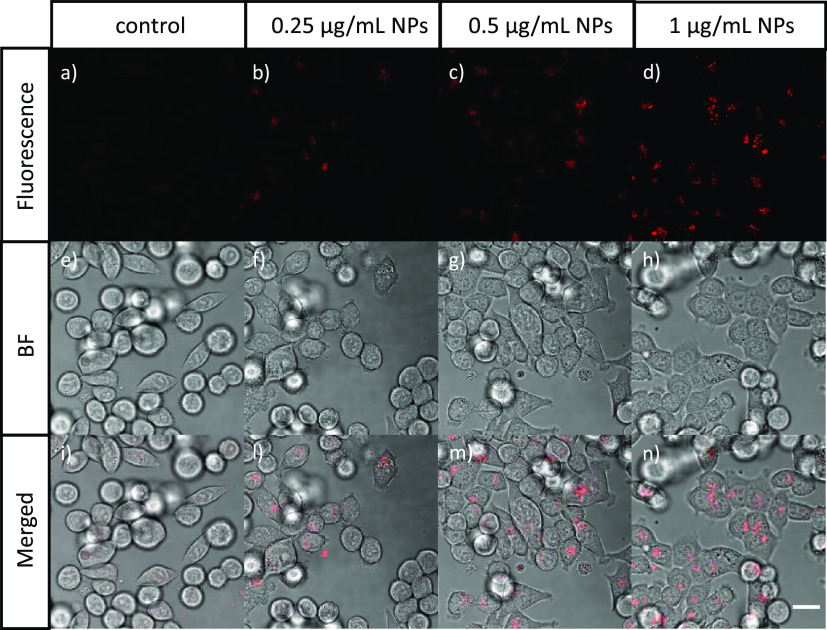
Confocal microscopy images in (a–d) fluorescence, (e–h) bright field, and (i–n) merged signals of HCT-116 cancer cells incubated with (a, e, i) 0 μg/mL, (b, f, l) 0.25 μg/mL, (c, g, m) 0.5 μg/mL, and (d, h, n) 1 μg/mL of pegylated Er:Y_2_O_3_ nanoparticles after 24 h of incubation. Scale bar: 20 μm.

The NPs internalization was verified at 24 h for each NPs concentration. Thus, the solutions of pegylated particles at different concentrations (0–1 μg/mL) were incubated for 24 h with HCT-116 cells and were then observed with a confocal microscope. The concentrations of the NPs in pellets were evaluated by ICP-MS. The fluorescence and bright field confocal microscope images of the control (cells without NPs) and of the cells incubated with NPs at different concentrations are reported in Figure 4. Figure 4a–d shows that the fluorescence increases as a function of the NPs concentration. The signals obtained at the lowest concentration were not very intense, and thus, no lower concentrations were explored. Furthermore, this fluorescence comes from inside the cells, indicating that an internalization has taken place.

**Figure 5:**
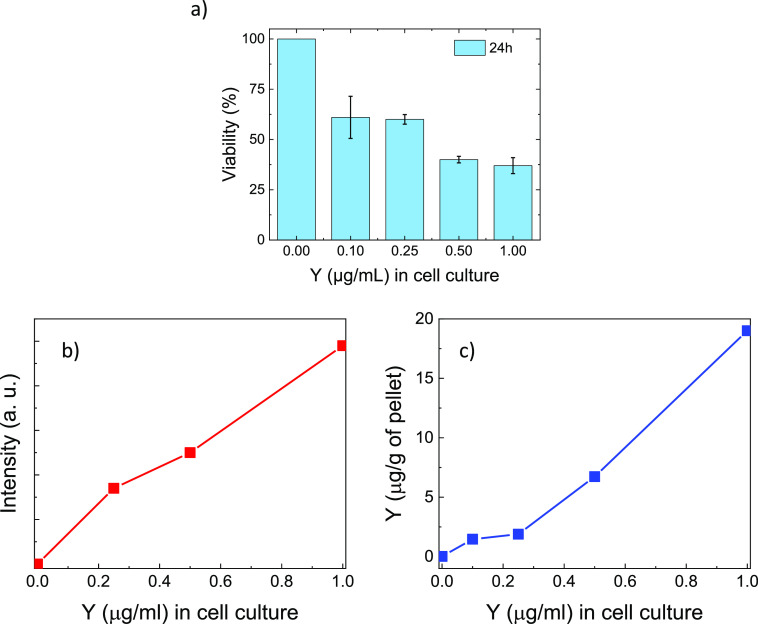
(a) Viability percentage (%) of HCT-116 incubated with pegylated Er:Y_2_O_3_ nanoparticles at different concentrations at 24 h of incubation. Values are reported as mean ± SD; *n* = 4. All data showed a p-value < 0.05 vs control. (b) Confocal microscopy photoluminescence intensity of pegylated Er:Y_2_O_3_ nanoparticles internalized in cells after incubation with different NPs concentrations. (c) Concentration of yttrium measured in pellets by ICP-MS versus concentrations of yttrium in the incubation medium. In all cases, the Y concentration in the cell culture is related to the NPs concentration.

The confocal fluorescence images were analyzed, as described in Materials and Methods, in order to quantify the increase in fluorescence as the concentrations of the incubated NPs solutions increased, and the results are reported in Figure 5b.

To evaluate and confirm the internalization of NPs into the cells, ICP measurements were performed to measure the amount of Y present in the cellular pellets obtained from incubation with NPs at different concentrations, and the results are shown in Figure 5c. Since the matrix of the nanoparticles is Y_2_O_3_, measuring the amount of Y, gives us an indirect measure of the amount of NPs. All the NPs present outside the cells were eliminated by washing, and thus, the concentration of Y present in the pellets was equivalent to the concentration of Y inside the cells, ultimately linked to the number of internalized NPs. The results (Figure 5c) show an increase in the concentration of Y present inside the pellets consistent with the increase of NP concentration in cell culture. Furthermore, the washing waters were also analyzed by the ICP, and no significant Y concentrations were found. It is noteworthy that the curve trend observed in Figures 5b and c is similar, indicating that there is an increase in the fluorescence signal as the amount of internalized NPs increases.

Combining the toxicity studies with the confocal fluorescence studies, it is possible to find the optimal concentration for in vivo labeling applications for which nontoxic NPs, with a fluorescence signal high enough to be detectable at the confocal microscope, are obtained; this concentration is around 0.25 μg/mL for the type of NPs presented in this work.

**Figure 6:**
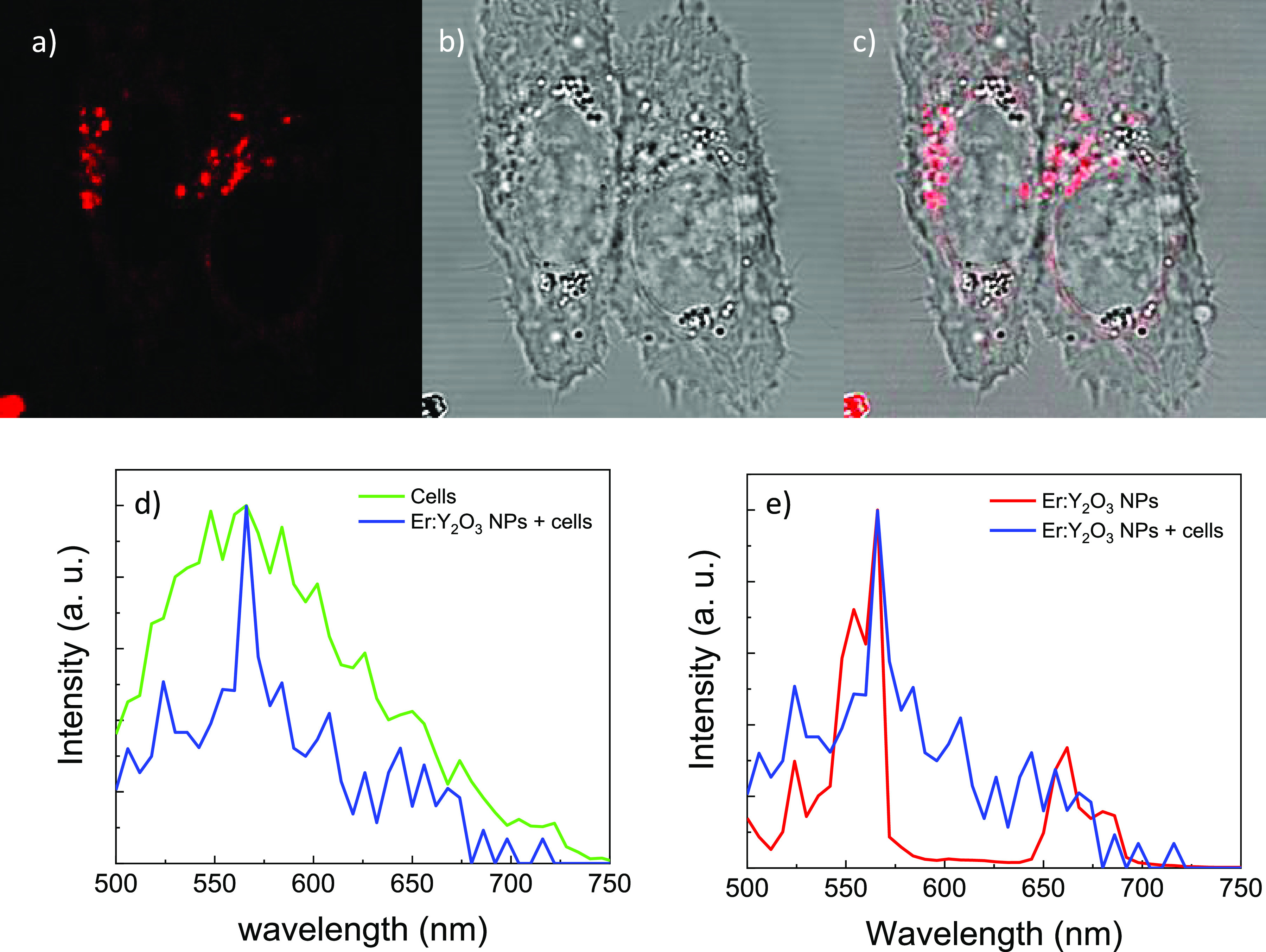
Confocal microscopy micrographs in (a) fluorescence, (b) bright field, and (c) merged of HCT-116 cancer cells incubated with 1 μg/mL of pegylated Er:Y_2_O_3_ nanoparticles. Photoluminescence spectra obtained by confocal microscopy for (d) cells and cells incubated with pegylated nanoparticles and (e) pegylated Er:Y_2_O_3_ nanoparticles alone and pegylated Er:Y_2_O_3_ nanoparticles and cells.

The cells were also observed with higher magnification through the confocal microscope, in order to localize the fluorescence signal, and the results are shown in Figure 6a–c. The fluorescence appears to be localized inside the cells but still outside the cell nucleus. Furthermore, the fluorescence spectra of either the cells alone (background signal) or the cells incubated with the NPs were obtained by confocal microscopy (Figure 6d). The two spectra overlap, even though the shape of the peak in the two cases is totally different, showing a very broad peak in the case of cellular autofluorescence, and sharp peaks at 523 and 565 nm in the case of cells in the presence of NPs. These peaks coincide with the peaks of the NPs alone (Figure 6e), even though a background signal linked to the self-fluorescence of cells is present.

## 4 Conclusions

In conclusion, in this study, luminescent Er-doped Y_2_O_3_ nanoparticles were successfully synthesized and characterized, highlighting their potential for in vivo bioimaging applications. The nontoxic nature of these nanoparticles, along with their efficient internalization within cells, supports their suitability as biocompatible probes for biomedical research. The optimal nontoxic concentration of approximately 0.25 μg/mL for in vivo bioimaging ensures a detectable fluorescence signal, further solidifying their suitability in this context.

The combined approach that made use of two very different techniques, i.e., confocal microscopy that allows us to obtain the spatial localization of NPs inside the cells and ICP-MS that is able to single out the contribution of the material under study, provides a powerful tool in the study of biological systems.

These findings underscore the versatility of Er-doped Y_2_O_3_ nanoparticles as promising candidates for targeted drug delivery and bioimaging applications for potential advancement of personalized medicine and therapeutic interventions in various diseases. Moreover, the functionalization of these nanoparticles with specific antibodies presents an innovative approach for developing optical sensors, enabling selective isolation and comprehensive characterization of specific extracellular vesicles in different cancer types, which could enhance the specificity and efficacy of liquid biopsies.

The future perspective is that these Er-doped Y_2_O_3_ nanoparticles could pave the way for the creation of extremely useful tools in the field of biomedical sciences, contributing to advancements in healthcare, potentially improving patient outcomes, and facilitating precise and personalized medical interventions.
